# Neuropathic Pain: A Comprehensive Bibliometric Analysis of Research Trends, Contributions, and Future Directions

**DOI:** 10.1007/s11916-025-01384-1

**Published:** 2025-04-04

**Authors:** Matteo Luigi Giuseppe Leoni, Marco Mercieri, Omar Viswanath, Marco Cascella, Martina Rekatsina, Alberto Pasqualucci, Annalisa Caruso, Giustino Varrassi

**Affiliations:** 1 Department of Medical and Surgical Sciences and Translational Medicine, Sapienza University of Rome, Rome, Italy; 2 Department of Anesthesiology, Creighton University School of Medicine, Phoenix, AZ USA; 3 Department of Medicine, Surgery and Dentistry, University of Salerno, Baronissi, Italy; 4 Department of Anaesthesia and Pain Management, National and Kapodistrian University of Athens, Athens, Greece; 5 Anaesthesiology and Critical Care, University of Perugia, Perugia, Italy; 6 Department of Surgery, ASST Lodi, Lodi, Italy; 7 Fondazione Paolo Procacci, 00193 Rome, Italy

**Keywords:** Neuropathic pain, Bibliometric analysis, Pain management, Chronic pain, Research trends, Neurostimulation, Pain research

## Abstract

**Background:**

Neuropathic pain represents a significant public health concern due to its complex pathophysiology and the disability it can cause. Despite advancements in understanding its underlying mechanisms and potential treatments, challenges persist in achieving effective management. This bibliometric analysis aims to offer a comprehensive overview of research trends, key contributors, and existing gaps in the literature on neuropathic pain, providing valuable insights to guide future studies and enhance clinical approaches.

**Methods:**

A bibliometric analysis was conducted using the Web of Science Core Collection (WoSCC) database. Key metrics, including publication trends, citation patterns, co-authorship networks, and keyword co-occurrence, were evaluated. Statistical analyses included average annual percentage change (APC) assessments and trend forecasting with an Auto Regressive Integrated Moving Average (ARIMA) model.

**Results:**

A total of 9,974 studies published between 2005 and 2024 were included. Publications peaked between 2021 and 2022 but showed a slight decline thereafter, with forecasts predicting a steady increase from 2025 to 2030. Most papers were published in high-impact Q1 journals, reflecting the quality of research. Co-authorship analysis revealed central hubs of collaboration in the USA and China, with limited integration of smaller countries into the global research network. Keyword analysis identified multiple thematic clusters, including "chronic pain," "molecular mechanisms," and "clinical management." Specific gaps were noted in understanding personalized therapeutic approaches, and non-pharmacological interventions.

**Conclusions:**

This analysis underscores the critical need for continued research to address gaps in diagnosis, treatment, and management of neuropathic pain. Strengthening international collaborations and fostering multidisciplinary efforts will be pivotal in advancing this field.

## Background

Neuropathic pain, as defined by the International Association for the Study of Pain (IASP), is caused by a lesion or disease of the somatosensory nervous system [1]. Its prevalence is estimated to range between 7 and 10% in population-based studies, highlighting its substantial public health impact [2, 3]. Patients with neuropathic pain often experience symptoms such as burning sensations, shooting pain, and allodynia, which are frequently resistant to standard analgesics [4]. Neuropathic pain is particularly challenging to manage due to its complex pathophysiology, which involves both peripheral and central mechanisms contributing to pain sensitization and chronicity [5, 6]. Common causes of neuropathic pain include diabetes, postherpetic neuralgia, spinal cord injuries, and chemotherapy-induced neuropathy [7]. All these conditions significantly impair quality of life and imposes a heavy economic burden on healthcare systems [8].

Recent years have seen considerable progress in understanding the underlying mechanisms of neuropathic pain [9, 10]. Emerging evidence suggests that even gut microbiota plays a role in the pathophysiology of neuropathic pain through its interactions with immune mediators, metabolites, and the nervous system, which encompass both central and peripheral pathways [11]. In addition to a deeper mechanistic understanding, innovative pharmacological and non-pharmacological therapies have been studied, offering new hope for managing neuropathic pain [12, 13]. Among these, palmitoylethanolamide (PEA) has gained attention for its anti-inflammatory and analgesic properties, resulting as a promising therapeutic option [14].

Despite advancements in understanding its mechanisms, there remains a critical need for further research to improve diagnosis, develop more effective therapies, and optimize management strategies for neuropathic pain [15]. In fact, epidemiological studies revealed that a significant number of patients with neuropathic pain failed to receive adequate treatment [16, 17]. This gap can be attributed to several factors, including challenges in achieving accurate diagnoses, limited comprehension of the underlying pathophysiological mechanisms, and the relative ineffectiveness of treatments [18].

Given these challenges, we conducted a comprehensive bibliometric analysis to deepen the understanding of neuropathic pain research. This approach enables the identification of prominent authors, leading institutions, and pivotal research areas, providing a clearer picture of the evolution of scientific knowledge in this field [19]. Moreover, our analysis aims to highlight existing gaps in literature, offering critical insights to inform and direct future research efforts.

## Methods

### Search Strategy and Data Collection

Our study employed a robust methodology aligned with established practices in bibliometric research [20, 21]. Bibliometrics quantitatively analyzes academic literature, offering insights into research trends, key contributors, and the evolution of scientific knowledge. To identify relevant publications, we used the Web of Science Core Collection (WoSCC) database, leveraging targeted search queries to comprehensively review articles on neuropathic pain. The search strategy included the following: (TI = (neuropathic AND pain)) AND ((DT = = ("ARTICLE" OR "REVIEW" OR "PROCEEDINGS PAPER" OR "EDITORIAL MATERIAL" OR "LETTER")). The search was restricted to English-language publications, excluding book chapters and congress abstracts. Data collection was completed in January 2025. The dataset underwent meticulous cleaning to eliminate duplicates and irrelevant entries, ensuring reliability and accuracy. Exported data were saved in TXT format for "full records and references" and Microsoft Excel (.xlsx) format to facilitate further analysis. Key bibliometric indicators, such as journal quartile rankings (Q) and impact factors (IF), were sourced from the 2023 Journal Citation Reports™ (Clarivate). The titles and abstracts of the retrieved articles were independently screened by three authors (A.P., A.C., and M.R.), with full-text versions reviewed for comprehensive evaluation. No articles were excluded during this process. To ensure data accuracy, all records were cross-checked, and discrepancies were resolved through discussions involving the first and last authors (M.L.G.L. and G.V.). This systematic approach ensured a high-quality dataset for analyzing patterns, assessing impact, and tracing the progression of research in neuropathic pain.

### Data Processing and Analysis

The number of publications and citations related to neuropathic pain was quantitatively assessed using the Citation Report tool from Clarivate Analytics. For network analysis, VOSviewer (version 1.6.20, Leiden University, Leiden, the Netherlands) was employed to generate visual maps depicting the relationships between authors, institutions, and research topics. This allowed for the identification of key patterns, emerging trends, and collaborative clusters within the scientific literature [22]. To evaluate trends in publication and citation patterns over time, the Dickey-Fuller test was applied to determine whether the data exhibited stationarity or were influenced by an underlying trend [23]. The average annual percentage change (APC) in publication output was calculated by applying a linear regression model to the logarithmic transformation of yearly publication counts. This method provided a statistical evaluation of the growth rate and overall trend. Furthermore, join-point regression analysis was performed to identify significant shifts in publication trends during the study period, offering a more detailed understanding of temporal changes [24]. To forecast future research output on neuropathic pain, an Auto Regressive Integrated Moving Average (ARIMA) model was applied, projecting publication numbers for the years 2025 to 2030. All statistical analyses and modelling were conducted using R software v4.3.2 (R Foundation for Statistical Computing, Vienna, Austria, www.r-project.org).

## Results

**Fig. 1:**
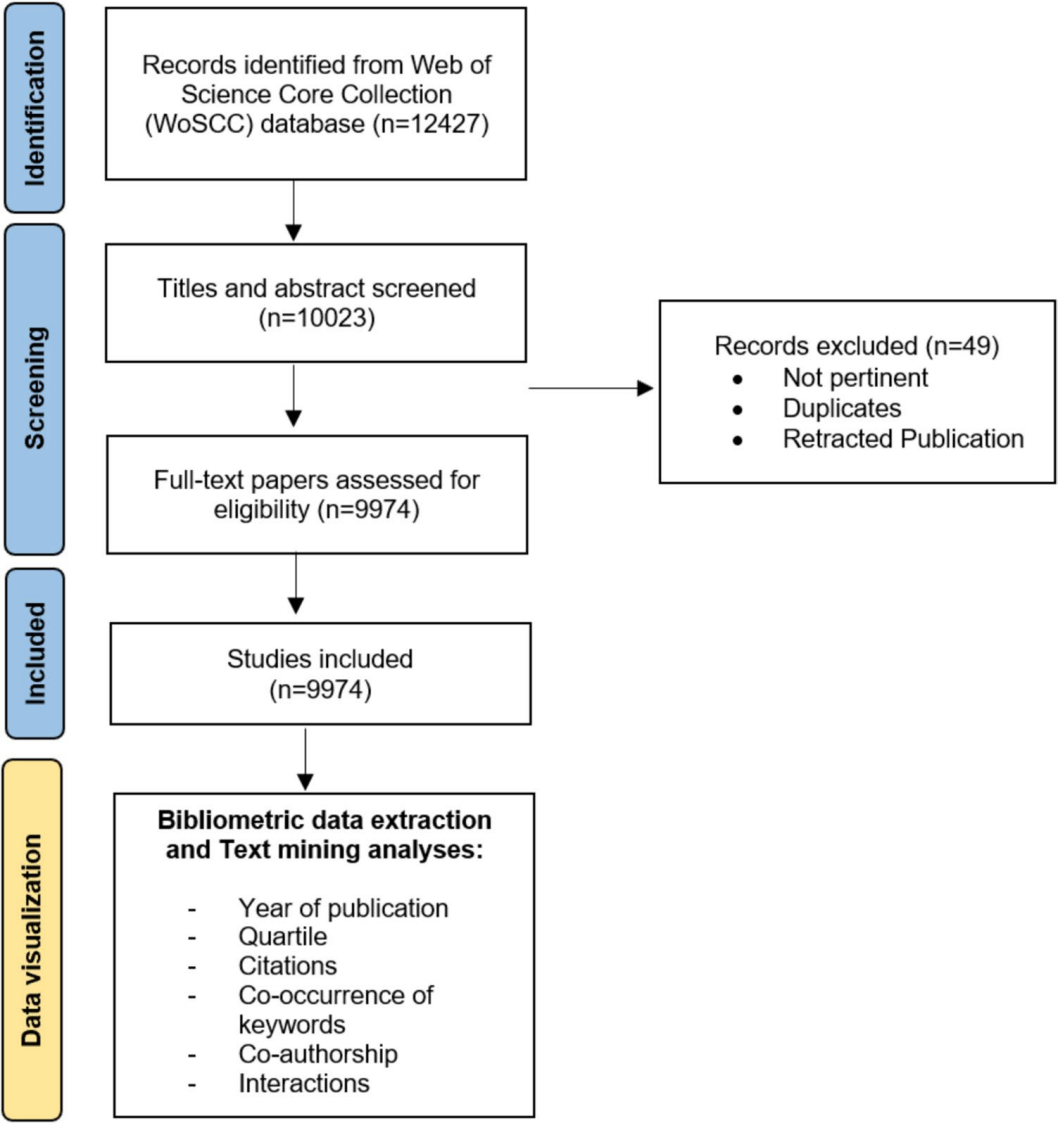
Flowchart of the study

A total of 12,427 records were initially identified from the WoSCC database. Following the screening process, which included the removal of duplicates, non-pertinent studies and retracted publications, 9,974 studies were deemed eligible for analysis (Fig. 1). The selected studies underwent bibliometric data extraction. Key aspects analyzed included the year of publication, quartile rankings, citation counts, keyword co-occurrence patterns, co-authorship relationships, and international collaborations [25]. This approach provided comprehensive insights into the trends, research focus, and collaborative dynamics within the field of neuropathic pain research.

**Fig. 2:**
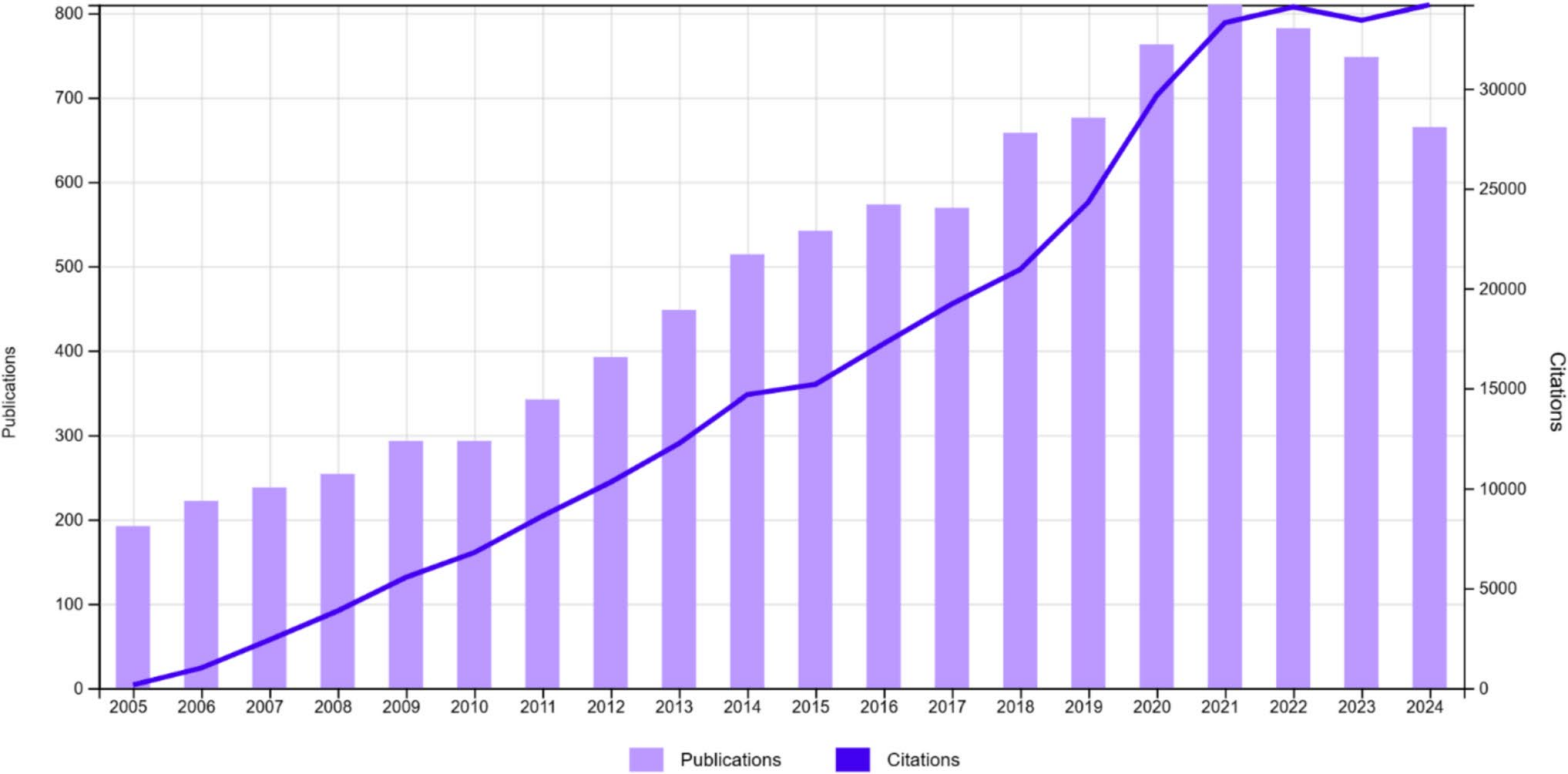
Annual publications and citations distribution

According to the Citation Report from WoSCC, the trend in scientific publications from 2005 to 2024 showed a significant growth, peaking around 2021–2022, followed by a slight decline (equation for the Number of publications = −68318.44 + 34.16 * Year; *p* < 0.001, Adjusted R^2^ = 0.93), (Fig. 2). The Dickey-Fuller test confirmed that the time series data was non-stationary (Dickey-Fuller = −1.8563, *p* = 0.6271), suggesting that its statistical properties vary over time.

**Fig. 3:**
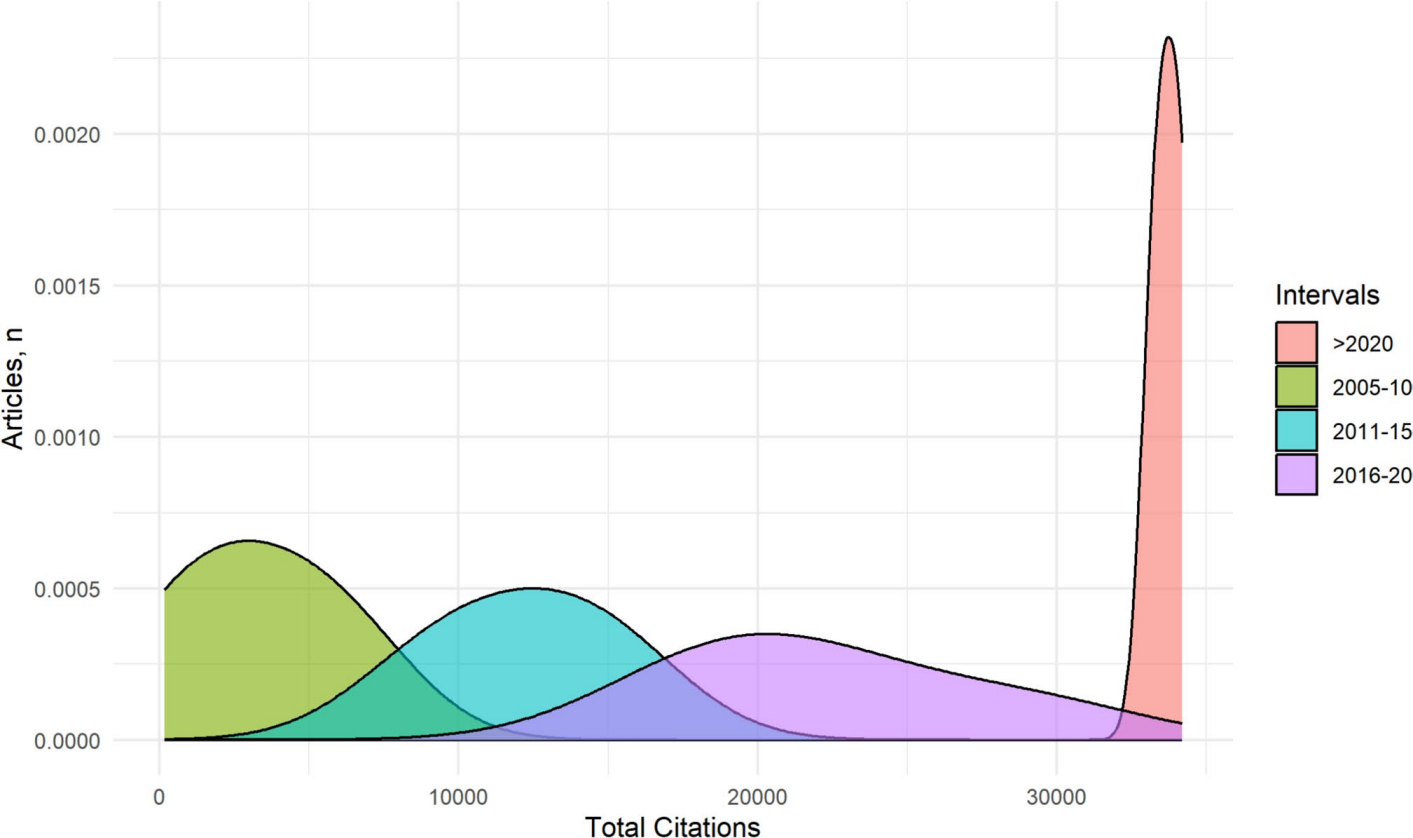
Citation analysis by time intervals. The density plot represents the distribution of total citations across four publication intervals: 2005–2010, 2011–2015, 2016–2020, and > 2020

Articles were cited 328,071 times with 32.9 mean citations per article. The density plot illustrated the distribution of total citations for articles grouped into four publication intervals: 2005–2010, 2011–2015, 2016–2020, and > 2020 (Fig. 3). The analysis revealed distinct trends in citation activity across these intervals. Articles published between 2005 and 2010 displayed moderate citation density, reflecting steady academic interest during that period. The citation density increased significantly for the 2011–2015 and 2016–2020 intervals, with a pronounced peak indicating heightened citation activity, hence high scientific interest. Articles published after 2020 exhibited the highest density, reflecting an intense focus on recent research. Overall, the results highlighted a progressive increase in citation intensity over time, with the most recent period dominating the citation landscape.

**Fig. 4:**
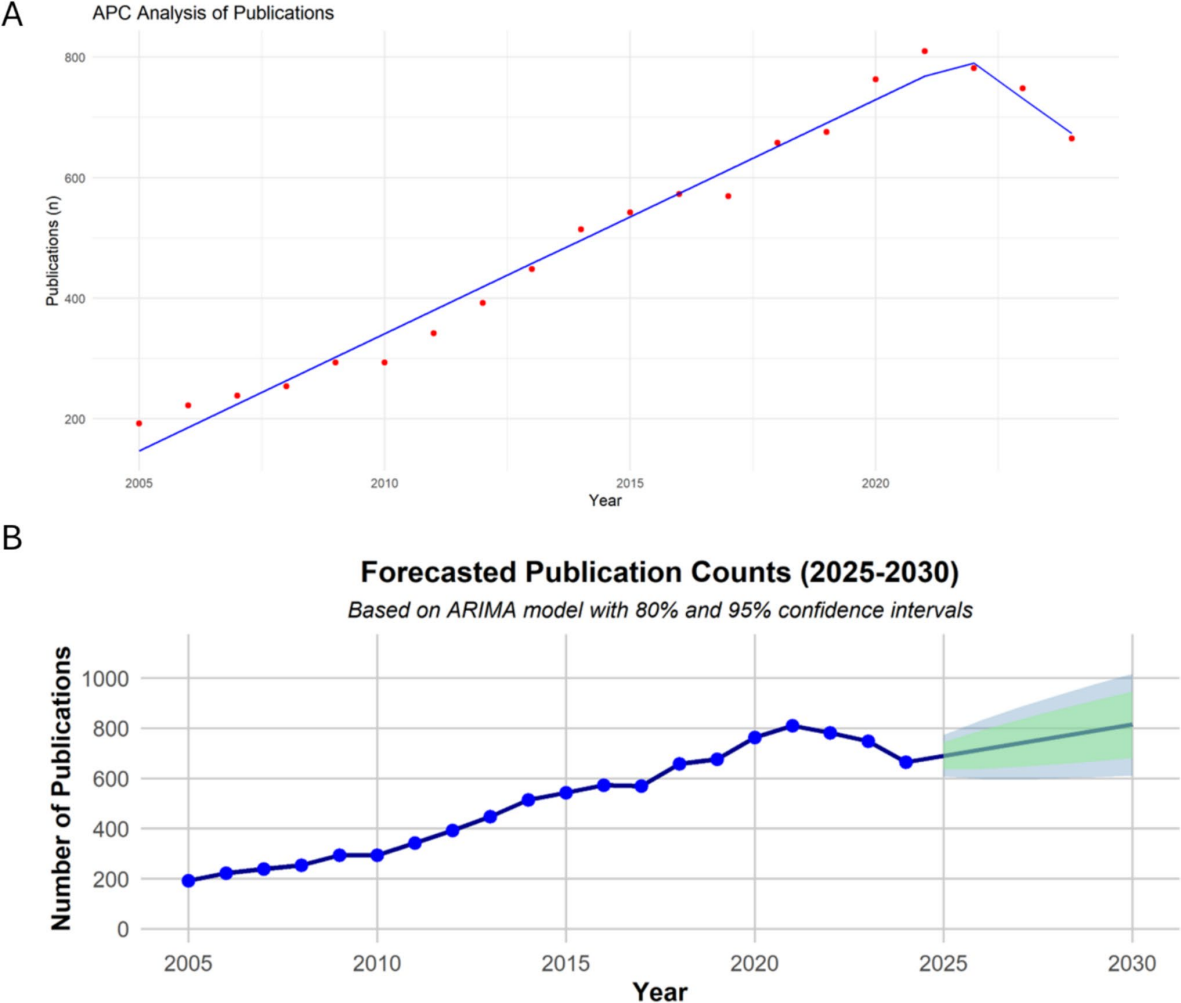
Trends and forecasts in publication activity. **A**) APC analysis of publications over the study period (2005–2024), showing a steady increase in publication activity until 2021, followed by a decline. **B**) Forecasted publication counts from 2025 to 2030 using an ARIMA model. The blue line represents the predicted annual number of publications, with shaded areas indicating the 80% (light green) and 95% (light blue) confidence intervals, reflecting a gradual upward trend and sustained interest in the research field

The APC analysis of publication trends (Fig. 4A) revealed significant changes over the study period. The join-point analysis showed a consistent increase in publication rates from 2005 to 2021, highlighting a growing interest in the research topic during this time (slope = + 20.2). However, after 2021, a decline in publication activity was observed, indicating a potential shift in research focus (slope = −26.3). The forecasted publication counts for 2025 to 2030, modelled using an ARIMA approach, provided valuable insights into future trends during this period (Fig. 4B). The projections indicated a slight upward trajectory, with point estimates increasing from 689.9 in 2025 to 814.4 in 2030. The relatively narrow confidence intervals, as shown by the shaded regions at 80% and 95%, emphasized the stability of this trend (80% confidence interval range: 635–910; 95% confidence interval range: 607–1017). These forecasts reflect sustained interest in the research field, with continued growth and minimal variability expected in the upcoming years.

The majority of the documents were published in prestigious Q1 journals, reflecting their high-quality contributions to the field. However, a notable portion of publications also appeared in Q3 journals, particularly within the domains of neurology and neuroscience. Among the selected papers, 81% were categorized as original research articles, while 13% comprised review articles. The ten journals with the highest number of publications on neuropathic pain are presented in Table 1.

**Table 1:** Leading ten Journals ordered by publication volume. This table highlights the journals with the highest number of publications related to neuropathic pain research

Journal nam	Documents *n* = 81, (%)	5-Year Journal Impact factor	Category quartile
Pain	565 (5.7%)	7.1	Q1
Molecular pain	205 (2.1%)	3.2	Q3
European Journal of Pain	198 (2%)	3.8	Q1
Neuroscience letters	168 (1.7%)	2.5	Q3
Journal of Pain Research	163 (1.6%)	2.8	Q3
Pain Medicine	162 (1.6%)	3.2	Q1
European Journal of Pharmacology	128 (1.3%)	4.3	Q1
Journal of Pain	125 (1.3%)	5.0	Q1
International Journal of Molecular Sciences	115 (1.2%)	5.6	Q2
Journal of Neuroscience	113 (1.1%)	5.3	Q1

**Fig. 5:**
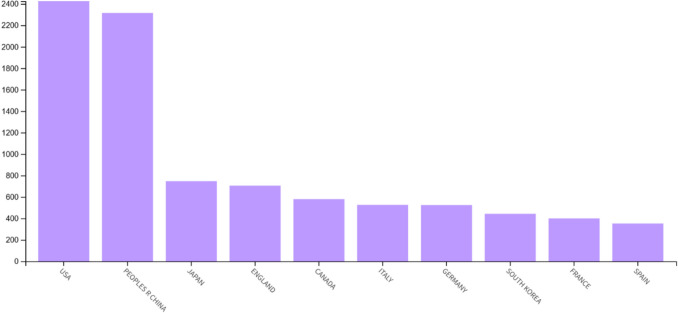
Geographical distribution of published papers. This figure illustrates the number of publications by country on neuropathic pain research, with the United States and China leading as the top contributors, followed by Japan, England, and other nations, showcasing varying levels of engagement in the field

The distribution of published papers across the top ten countries is illustrated in Fig. 5, showing their contributions to the field. The United States emerges as the leading contributor with the highest number of publications (24.3%), followed closely by China (23.2%), both reflecting their dominant role in advancing research. Japan (7.5%) and England (7.1%) form the next tier of contributors, demonstrating significant but slightly lower engagement. Countries such as Canada (5.8%), Italy (5.3%), and Germany (5.3%) also show notable involvement. South Korea (4.3%), France (4%), and Spain (3.5%) complete the list, indicating a relatively lower level of activity in publication output.

**Fig. 6:**
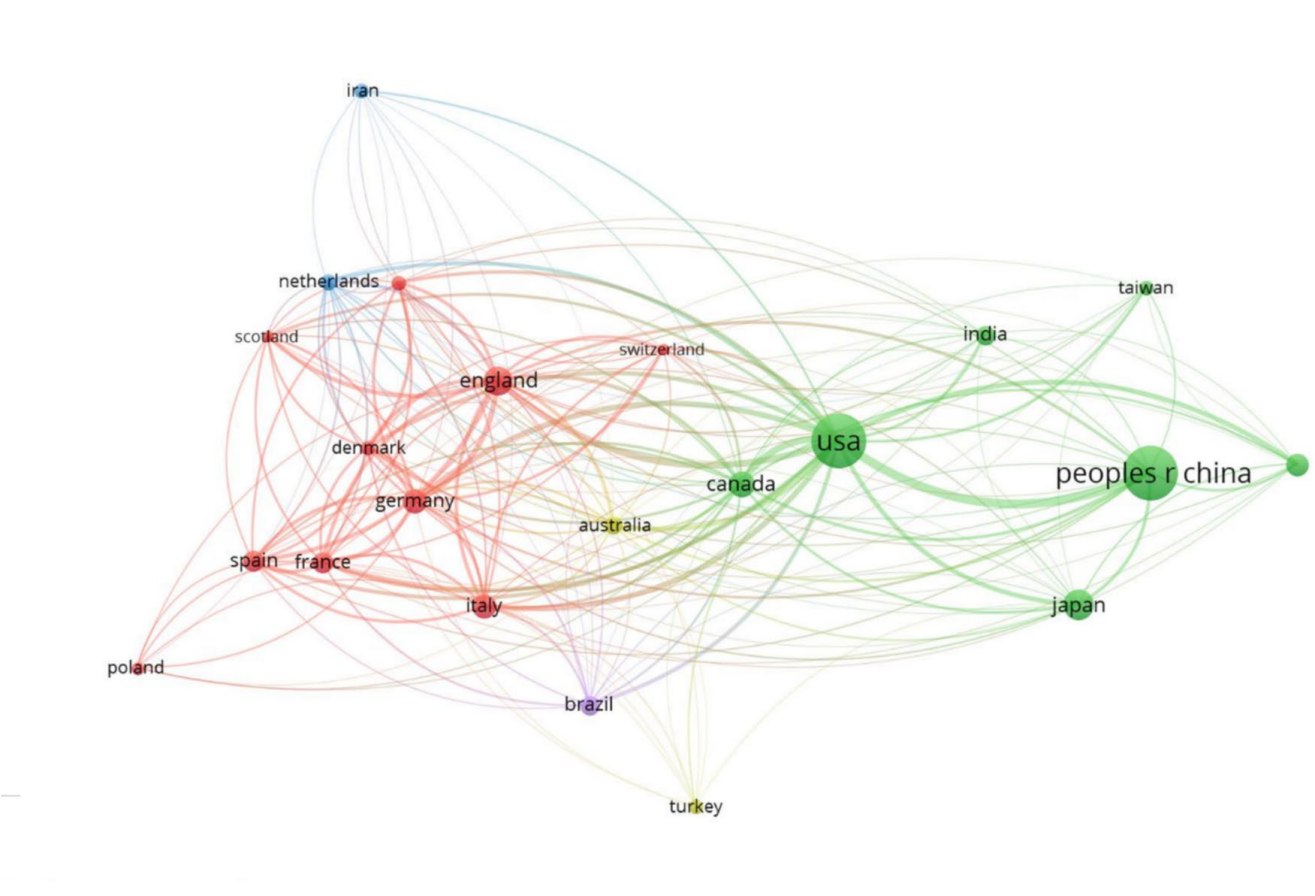
Co-authorship network among countries in neuropathic pain research. Nodes represent countries, with node size indicating the number of publications and connecting lines depicting collaborative relationships. Line thickness reflects the strength of these collaborations

The co-authorship analysis among countries, with a minimum threshold of 100 publications per country, provided insights into international collaboration in neuropathic pain research (Fig. 6). A total of 22 countries met this criterion, and the network revealed a dense and interconnected pattern of collaboration, with the United States and China emerging as central hubs, demonstrating active partnerships with several countries, including Canada, Japan, and India. European nations such as England, Germany, and The Netherlands exhibited strong intra-regional collaborations, forming a cohesive subnetwork. However, some countries, such as Poland and Brazil, appeared less integrated within the broader collaboration network. This analysis underscored the need to foster stronger connections among underrepresented countries to create a more unified global research community and to advance partnerships in neuropathic pain research.

**Fig. 7:**
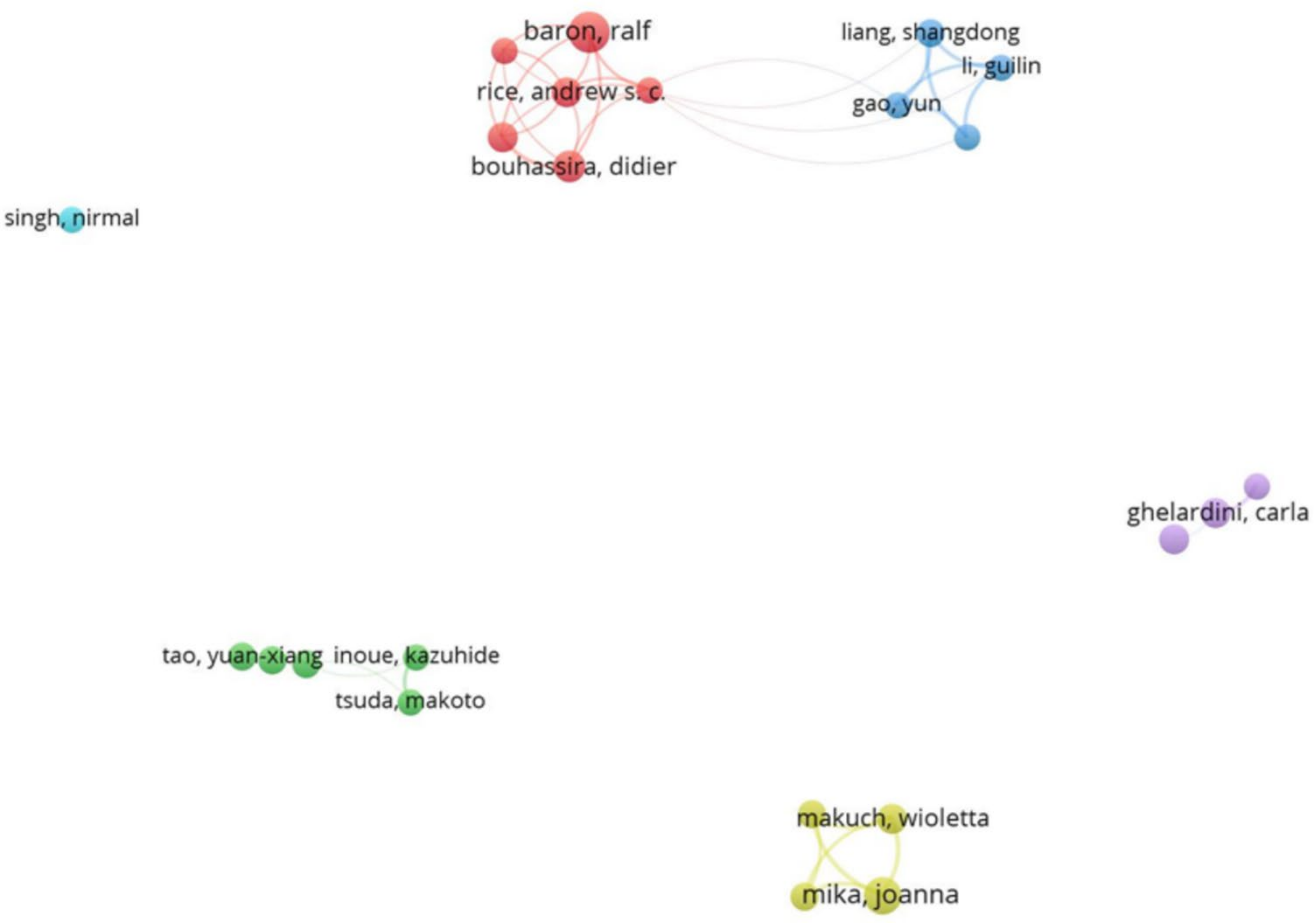
Co-authorship analysis of authors. This figure highlights the collaborative networks among researchers in neuropathic pain. Each node represents an author, and the connecting lines denote co-authorship relationships. The visualization reveals prominent clusters of closely connected researchers alongside smaller or isolated groups, reflecting varying levels of collaboration within the field

The co-authorship network map for authors, created with a minimum threshold of twenty-five documents per author, revealed distinct clusters of collaboration within the field (Fig. 7). Six key clusters emerged, each demonstrating varying levels of interconnectivity. The largest cluster included closely associated authors such as Ralf Baron from Christian-Albrechts-Universität Kiel, Germany, Andrew S.C. Rice from Imperial College London, United Kingdom, and current president of the IASP Society, and Didier Bouhassira from Paris-Saclay University, France, showcasing a highly collaborative network. Another prominent cluster, centered on Chinese authors such as Shandong Liang and Guilin Li from Nanchang University, China, demonstrated regional collaboration with limited integration into broader networks. Additional smaller clusters, including authors such as Kazuhide Inoue from Kyushu University, Japan, and Carla Ghelardini from the University of Florence, Italy, highlighted localized collaboration or independent research efforts. Another cluster was centered around Joanna Mika from the Maj Institute of Pharmacology, Polish Academy of Sciences, Poland. Conversely, Nirmal Singh from Punjabi University, India, conducted isolated research on neuropathic pain without developing significant international collaborations. This map underscored a fragmented yet diverse pattern of collaboration among contributing authors in this field.

**Fig. 8:**
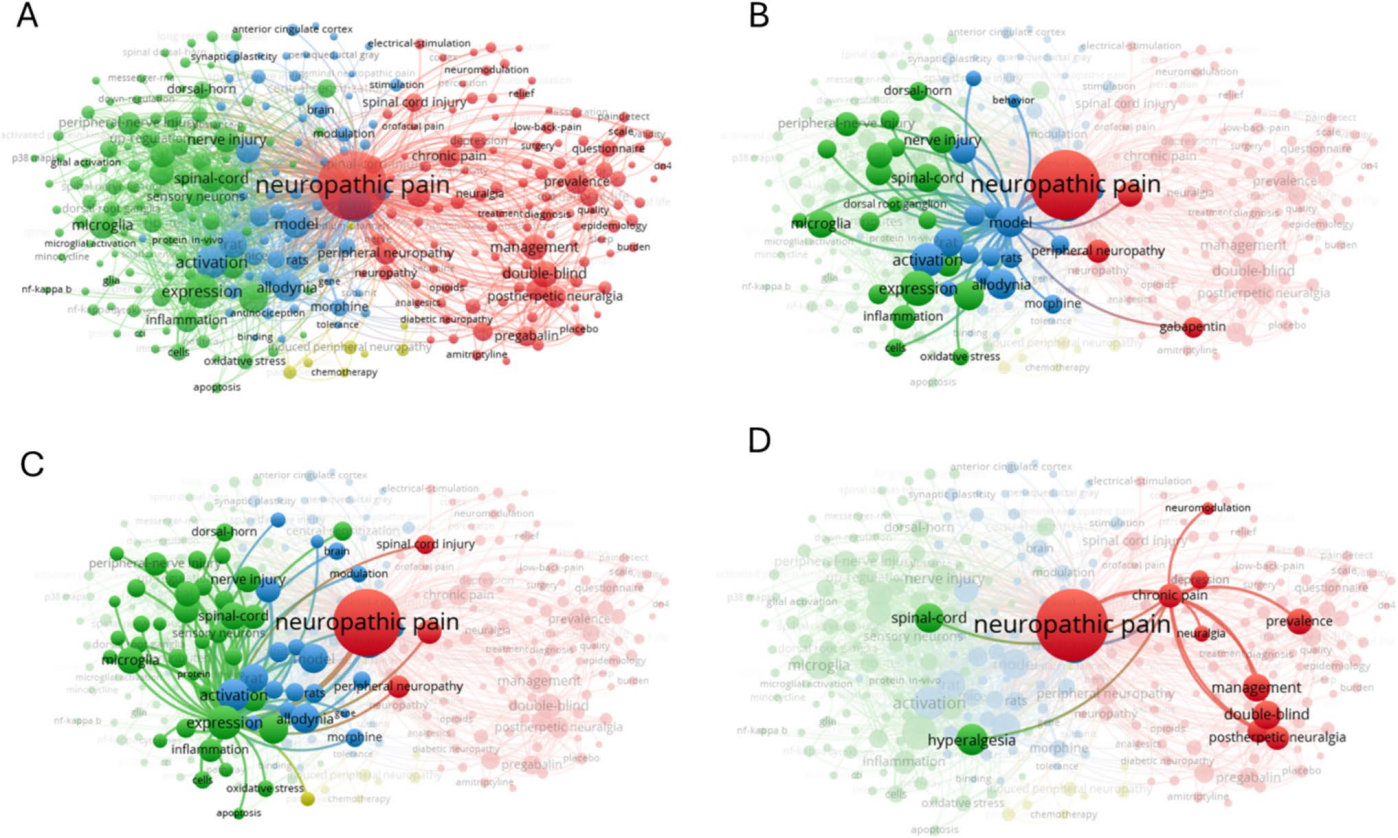
Keyword Co-Occurrence Network in Neuropathic Pain Research. **A**) The keyword co-occurrence analysis shows the relationships between frequently used terms in neuropathic pain research. Each node represents a keyword, with larger nodes indicating higher frequency in the dataset. Connections between nodes reflect co-occurrence within the same publications, with thicker lines representing stronger associations. This visualization highlights key research themes and their interconnections. To further explore specific subthemes within the network, additional focused analyses were performed and are shown in panels B, C, and D.** B**) emphasizes the use of "model", capturing studies involving animal models and experimental approaches to investigate neuropathic pain mechanisms, particularly those related to spinal cord and peripheral nerve injuries. **C**) focuses on "expression", showcasing molecular and cellular investigations into inflammation, oxidative stress, and microglial activation. **D**) centers on "chronic pain", highlighting research on neurostimulation, spinal cord injury, and the clinical aspects of pain management

The co-occurrence analysis of keywords, focusing on those appearing over fifty times in the dataset, identified several clusters representing major research areas in neuropathic pain (Fig. 8). The red cluster highlighted topics centered around "chronic pain," "prevalence," and "management," emphasized research on the epidemiology, treatment strategies, and patient management, including the role of medications such as gabapentin and amitriptyline. This cluster underscored the focus on double-blind studies and treatment efficacy in conditions such as postherpetic neuralgia and diabetic neuropathy. Due to the complexity of the data, additional keyword analyses were conducted to focus on specific subtopics (Fig. 8B, C, D). In Fig. 8B, the analysis centered on "model," showcasing research that utilized animal models and experimental studies to explore mechanisms of neuropathic pain, including spinal cord and peripheral nerve injuries. Another focus was shifted to "expression," highlighting molecular and cellular research on inflammation, oxidative stress, and the role of microglia in the pathophysiology of neuropathic pain (Fig. 8C). Finally, another topic was centered on "chronic pain," examining the interplay between neurostimulation, spinal cord injury, and clinical management (Fig. 8 D). This subgroup also explored patient outcomes, the role of electrical stimulation, and advances in non-invasive techniques for managing neuropathic pain. Together, these analyses highlighted the diversity of research efforts in neuropathic pain, encompassing experimental, molecular, and clinical dimensions.

## Discussion

This bibliometric analysis provides a comprehensive overview of the research landscape surrounding neuropathic pain, focusing on publication trends, collaborative networks, and emerging research themes. Our analysis identified a dynamic yet irregular trajectory in publication activity from 2005 to 2024. While periods of substantial growth were evident, such as the notable peak in publications between 2021 and 2022, these were followed by periods of decline, reflecting an inconsistent upward trend. This fluctuation likely mirrors the evolving priorities within neuropathic pain research, influenced by shifts in funding, advancements in related disciplines, and changes in clinical and scientific focus. This finding is not entirely novel, as a decline in the number of published papers after 2021 was also reported in a bibliometric analysis focusing on neuropathic pain in neurodegenerative diseases [26]. Additionally, the COVID-19 outbreak may have impacted the allocation of clinical resources and shifted the focus of published research topics [27]. However, the significant upward trend observed suggests that fundamental questions in neuropathic pain still need to be addressed. Despite advancements in scientific knowledge and the development of clinical applications, neuropathic pain remains a complex condition that is challenging to manage effectively. This persistent complexity highlights the necessity for ongoing and intensified research efforts, supported by substantial material and human resources, to enhance understanding and improve treatment outcomes. This need was further underscored by forecasting predictions, which indicate a consistently high and stable volume of publications over the next five years, reflecting sustained and growing interest in this field.

The distribution of studies across journals demonstrated considerable dispersion. While most documents were published in prestigious Q1 journals, underscoring their high-quality contributions to the field, a notable proportion appeared in Q3 journals, particularly within the domains of neurology and neuroscience. This pattern suggests that a significant portion of neuropathic pain research is conducted within these disciplines. However, it also highlights a potential gap in the allocation of space for neuropathic pain studies in highly impactful neurology-focused journals, indicating the need for greater recognition of the topic's importance in these outlets. *Pain* was the journal with the highest impact factor and the largest number of published papers on neuropathic pain. This finding underscores the journal's pivotal role in advancing neuropathic pain research, both in elucidating underlying mechanisms and in developing effective management strategies. Our findings also highlighted the importance of international collaboration in advancing neuropathic pain research. The co-authorship network revealed dense clusters of collaboration, particularly among countries such as the United States, China, and leading European nations. However, gaps remain in fostering partnerships with underrepresented regions, emphasizing the need for a more globally integrated approach.

If we assess the quality of articles based on citation counts, five articles stand out as the most highly cited. The article with the highest number of citations (*n* = 2053) was published by Finnerup et al. in 2015 [28]. This publication presents the revised Neuropathic Pain Special Interest Group (NeuPSIG) recommendations for the pharmacotherapy of neuropathic pain, which were updated following a systematic review and meta-analysis of 229 randomized, double-blind studies. It underscores the modest efficacy of existing treatments for neuropathic pain while strongly recommending tricyclic antidepressants, serotonin-noradrenaline reuptake inhibitors, pregabalin, and gabapentin as first-line therapies. Furthermore, it highlights a substantial unmet need due to inadequate treatment responses and limitations in clinical trials. The second most cited article (*n* = 1959) introduced the grading system (definite, probable, possible) for diagnosing neuropathic pain, developed by Treede et al. [29], which is based on neurological evidence and proposed for both clinical and research applications. Another highly cited article (*n* = 1933) by Rolke et al. [30] presented the standardized quantitative sensory testing (QST) protocol developed by the German Research Network on Neuropathic Pain (DFNS), which includes age- and gender-matched reference values to characterize somatosensory phenotypes in neuropathic pain patients, providing valuable insights into underlying mechanisms through precise sensory profiling. The fourth and fifth most highly cited articles (*n* = 1751 and 1630, respectively) were the paper presenting the development of the DN4 questionnaire by the French Neuropathic Pain Group [31] and the paper introducing the painDETECT screening tool [32], designed to identify neuropathic components in patients with low back pain.

The co-authorship analysis identified six distinct clusters, each exhibiting varying degrees of interconnectivity within the group but with no links connecting the clusters to one another. This finding underscores the presence of independent networks of researchers in the field, suggesting that each cluster operates as a separate aggregation of scientists with minimal collaboration across groups. Such a fragmented structure may indicate a lack of interdisciplinary exchange or communication between research teams, potentially limiting the broader dissemination of knowledge and innovation within the field. Encouraging stronger cross-cluster collaborations could foster a more integrated research community and promote a more unified approach to advancing the field. The largest cluster included Ralf Baron, Andrew S.C. Rice and Didier Bouhassira. One of the most influential articles from this group, cited nearly 600 times, described distinct neuropathic pain patterns using QST data from 902 patients (test cohort) and 233 patients (validation cohort) [33]. Through cluster analysis, three distinct sensory subgroups were identified. The first subgroup, characterized by sensory loss (42%), exhibited loss of small and large fiber’s function accompanied by paradoxical heat sensations. The second subgroup, representing 33% of the patients, was defined by thermal hyperalgesia, with preserved sensory function but heightened sensitivity to heat and cold, along with mild mechanical allodynia. The third subgroup, accounting for 24%, was characterized by mechanical hyperalgesia, involving small fiber loss, pinprick hyperalgesia, and dynamic mechanical allodynia. These subgroups were linked to specific etiologies, providing valuable insights into the relationship between sensory profiles and pathophysiological mechanisms. This classification has significant implications for clinical trial design, enabling the targeting of treatment-responsive populations and improving therapeutic outcomes.

The second cluster focused on Chinese authors, from Nanchang University, China. Their most prominent contribution to neuropathic pain research highlighted the role of adenosine triphosphate (ATP) as an extracellular signaling molecule acting via P2X and P2Y purinergic receptors expressed by satellite glial cells (SGCs) and macrophages [34]. They demonstrated how these cells, forming a macrophage-SGC-neuron triad in response to injury, release inflammatory cytokines and pro-nociceptive mediators, which enhance neuronal excitability and perpetuate inflammation-related neuropathic pain. A smaller cluster included Ghelardini C., from the University of Florence, Italy. Her scientific production on neuropathic pain was mainly focused on chemotherapy-induced neurotoxicity [35, 36] and diagnostic biomarker of peripheral neurotoxicity [37].

Another significant finding from our analysis was the identification of principal clusters derived from keyword co-occurrence analysis. These clusters offer valuable insights into the primary research themes and focal points within the field of neuropathic pain. By grouping frequently co-occurring keywords, the analysis highlights the interconnected areas of interest. The first identified cluster in the keyword co-occurrence analysis was centered on terms like "chronic pain," "prevalence," and "management," reflecting a strong research focus on understanding the treatment of neuropathic pain along with the evaluation of its real prevalence. Additionally, this cluster highlighted the prevalence of double-blind studies aimed at evaluating treatment efficacy, underscoring the commitment to evidence-based approaches in addressing these debilitating conditions. The keyword co-occurrence analysis revealed additional clusters that highlight the wide scope of research efforts in neuropathic pain. One cluster was centered on "model," emphasizing the use of animal models and experimental studies to investigate the mechanisms of neuropathic pain, including insights into spinal cord and peripheral nerve injuries. The most cited article (*n* = 527) on this topic, published by Nirmal Singh's group, provided a comprehensive review of approximately 40 animal models of neuropathic pain [38]. The study discussed their methodologies, behavioral characteristics, limitations, and advantages. These models have been instrumental in elucidating the peripheral and central mechanisms underlying chronic neuropathic pain and have significantly contributed to the development of novel therapeutic agents. However, the authors emphasized the importance of interpreting results within the specific context of each model, as variations in methodology can lead to significant differences in outcomes.

Another identified cluster centered on "expression," highlighting molecular and cellular research aimed at revealing the mechanisms underlying inflammation, oxidative stress, and the role of microglia and other critical proteins in the pathophysiology of neuropathic pain. According to the most cited article on this topic [39] (*n* = 209), microglia play a pivotal role in the development and persistence of neuropathic pain. Microglial activation is initiated in response to nerve injury, during which mediators such as Neuregulin-1, matrix metalloproteinases (MMP-2 and MMP-9), chemokine ligand 2 (CCL2), and fractalkine are released, further triggering and sustaining microglial activation. Activated microglia, in turn, release pro-inflammatory cytokines such as interleukin (IL)−6, IL-1β, and tumor necrosis factor-α (TNF-α), which enhance neuronal excitability and reduce inhibitory signals, contributing to the painful symptoms of neuropathic pain.

Another significant area of exploration highlighted the therapeutic potential of neuromodulation as a minimally invasive approach for managing neuropathic pain. This spectrum of interventions ranges from simpler techniques, such as pulsed radiofrequency (PRF) applied directly to targeted nervous tissues like the dorsal root ganglion (DRG) [40], to more advanced systems, including spinal cord stimulation (SCS) [41, 42] and dorsal root ganglion stimulation (DRGS) [43, 44]. However, the interconnected but distinct nature of the research clusters underscores the need for integrating findings across these domains to deepen the understanding and enhance the treatment of neuropathic pain. Integrating experimental, molecular, and clinical findings will be crucial for advancing the development of targeted interventions aimed at improving the quality of life for patients with neuropathic pain.

### Suggestions for Future Research

The main goal of a bibliometric study is to offer insights and strategic recommendations that can help shape and direct future research initiatives. Consequently, future research in neuropathic pain should focus on developing innovative and tailored therapeutic approaches to address the limitations of current treatments. Several agents, such as botulinum toxin A (BTX-A), ketamine, and mexiletine, have emerged as promising candidates for targeting specific pathways implicated in neuropathic pain. BTX-A is hypothesized to exert its analgesic effects through central mechanisms involving retrograde axonal transport [45]. It is now generally recommended as a third-line therapy for peripheral neuropathic pain due to its ability to modulate pain signals effectively. Mexiletine, an older anti-arrhythmic medication with sodium channel-blocking properties, has been repurposed for conditions such as primary erythromelalgia, a genetic disorder characterized by intense burning pain and vasomotor disturbances exacerbated by heat [46, 47]. This condition is associated with a gain-of-function mutation in Nav1.7 sodium channels, making mexiletine an effective therapeutic option [48]. Ketamine, an NMDA antagonist with potent analgesic properties, has demonstrated a statistically significant reduction in pain intensity when added to standard neuropathic pain treatments, both one week and 30 days after treatment. However, this benefit is counterbalanced by potential adverse effects, including increased discomfort and psychedelic symptoms [49]. Another notable candidate is ambroxol, traditionally used for its antitussive and anti-secretory effects. Ambroxol exhibits potent sodium channel-blocking activity by targeting Nav1.7 and Nav1.8 channels, coupled with anti-inflammatory properties [50]. These characteristics highlight its potential as a topical formulation for neuropathic pain management, offering a novel therapeutic option for patients.

Advances in neurostimulation techniques, including SCS, DRGS, and transcranial magnetic stimulation (rTMS), offer minimally invasive alternatives that act on central and peripheral mechanisms of pain. These modalities should be further refined through rigorous clinical trials to optimize stimulation parameters and expand their applications. Additionally, individualized therapeutic strategies based on patient stratification using biomarkers, sensory phenotyping, and genetic profiling can enhance treatment outcomes. Stratified approaches, as endorsed by regulatory guidelines, could link specific neuropathic pain profiles to targeted therapies, improving the precision of clinical trials and therapeutic interventions.

**Fig. 9:**
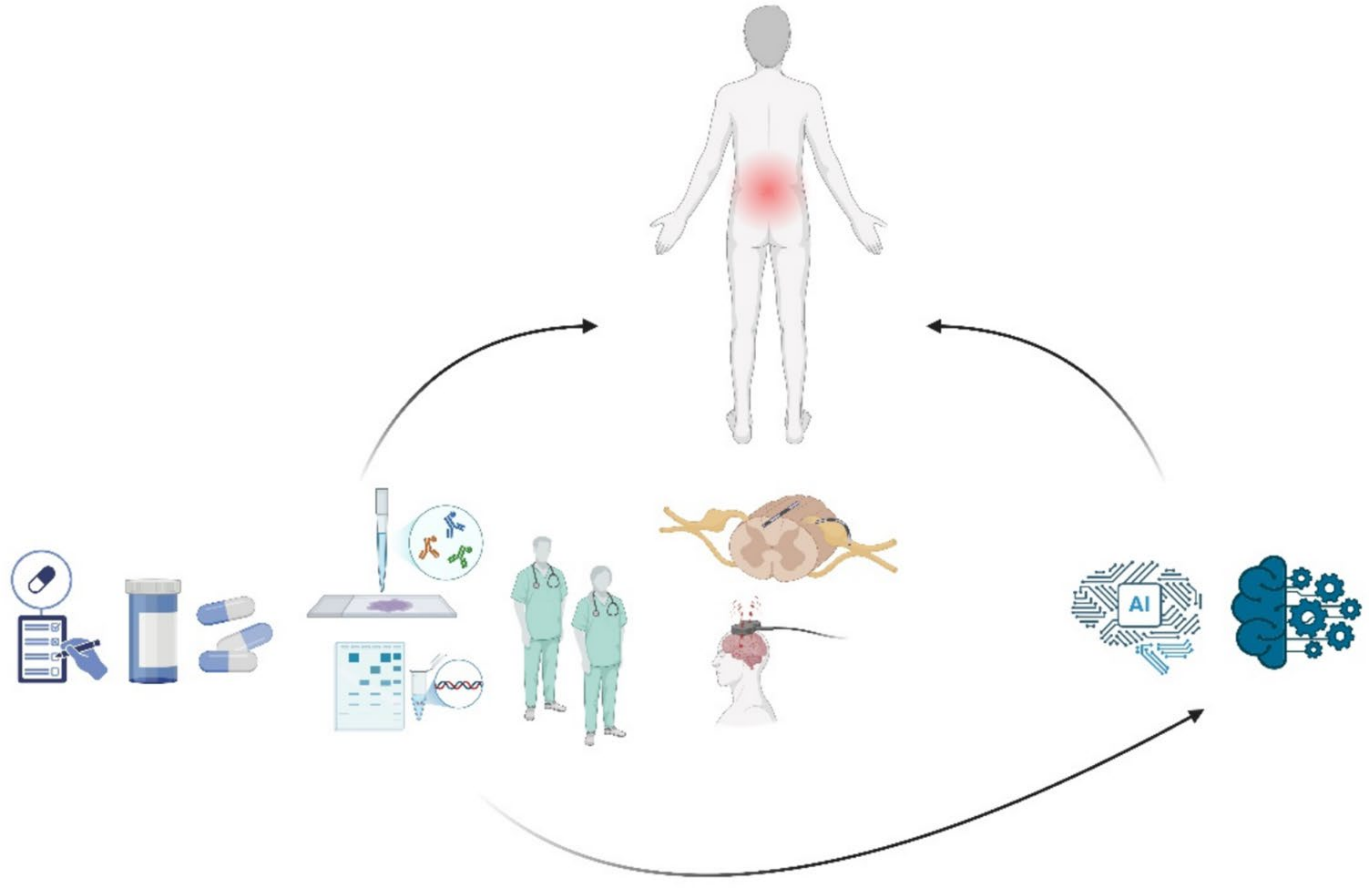
Proposed strategy for effective neuropathic pain management. This figure illustrates a comprehensive approach integrating new drug development, personalized therapeutic strategies based on patient stratification through biomarkers, sensory phenotyping, and genetic profiling. It emphasizes the importance of clinical data integration, advancements in neurostimulation techniques, and the application of artificial intelligence (AI) and machine learning (ML) models to optimize treatment outcomes for patients with neuropathic pain. Created with BioRender.com

Emerging technologies, such as artificial intelligence (AI) and machine learning (ML), offer significant potential for analyzing large datasets and identifying patterns in treatment responses, thereby advancing the personalization of care in pain medicine [51]. These advancements are particularly promising for the minimally invasive management of neuropathic pain, where AI could optimize procedural strategies and enhance treatment outcomes [52]. However, while the integration of clinical data with AI systems holds tremendous potential, several ethical challenges, including data privacy, algorithmic transparency, and equitable access, must be addressed before such approaches can be broadly implemented in clinical practice [53]. Finally, fostering interdisciplinary collaborations and addressing existing research gaps in future studies will likely lead to the development of more effective and comprehensive therapeutic strategies for this challenging condition. A graphical representation of the proposed strategy to enhance both research studies and clinical management of neuropathic pain is illustrated in Fig. 9.

### Limitations

Our bibliometric analysis has several limitations. First, the study was confined to articles retrieved exclusively from the WoSCC database, potentially excluding relevant studies indexed in other databases such as Scopus or PubMed, as well as grey literature sources. While WoSCC was selected for its reliability and widespread use in bibliometric research [54], this choice may have limited the comprehensiveness of our dataset. Second, bibliometric analyses inherently differ from systematic reviews or meta-analyses. Their purpose is to explore publication patterns, citation networks, and collaborative trends rather than to evaluate the quality or outcomes of specific studies [55]. Therefore, while our analysis provides a high-level overview of research trends in neuropathic pain, it does not replace in-depth evaluations of individual studies or treatment outcomes. Another limitation lies in the search strategy, which, despite being carefully designed, may not have fully captured all relevant publications on neuropathic pain. Although our comprehensive search initially identified a large volume of articles, many were excluded for irrelevance or duplication. This suggests that while the search strategy was effective in covering a significant portion of the indexed literature, some studies of importance might have been overlooked. Finally, temporal trends in publication activity may be influenced by external factors, such as variations in funding availability, regional research priorities, and evolving scientific interests. These factors could affect the representation of certain topics or geographic regions within the dataset. Additionally, since research is dynamic, bibliometric analyses should be periodically updated to reflect the latest developments in the field.

## Conclusions

This bibliometric analysis provides a comprehensive overview of the research landscape surrounding neuropathic pain, highlighting key trends, influential contributors, and existing gaps. Despite the significant burden neuropathic pain places on patients and healthcare systems, research in this field remains fragmented, with varying publication trends and limited international collaboration. The analysis revealed a focus on critical areas, including sensory profiling, the role of microglia in neuroinflammation, and the potential of innovative treatments such as neuromodulation and drug repositioning. These findings underscore the complexity of neuropathic pain, driven by diverse pathophysiological mechanisms and heterogeneous patient profiles. However, addressing gaps in global collaboration and fostering partnerships among underrepresented regions will be critical to advancing the field.

## Data Availability

The data used in this study were retrieved from the Web of Science Core Collection (WoSCC) database. Data supporting the findings of this study are available upon reasonable request from the corresponding author.

## Abbreviations

*AI*: Artificial Intelligence
*APC*: Average Percentage Change
*BTX-A*: Botulinum Toxin A
*CCI*: Chronic Constriction Injury
*CCL2*: Chemokine Ligand 2
*DRG*: Dorsal Root Ganglion
*ERK*: Extracellular Signal-Regulated Kinase
*IF*: Impact Factor
*IL*: Interleukin
*ML*: Machine Learning
*MMP*: Matrix Metalloproteinase
*Nav*: Voltage-Gated Sodium Channel
*PI3K*: Phosphatidylinositol 3-Kinase
*PRF*: Pulsed Radiofrequency
*Q*: Quartile
*QST*: Quantitative Sensory Testing
*RCT*: Randomized Controlled Trial
*Rtms*: Repetitive Transcranial Magnetic Stimulation
*SCS*: Spinal Cord Stimulation
*SGCs*: Satellite Glial Cells
*TENS*: Transcutaneous Electrical Nerve Stimulation
*tDCS*: Transcranial Direct Current Stimulation
*TNF*-α: Tumor Necrosis Factor-Alpha
*WoSCC*: Web of Science Core Collection
